# Impaired Brain Incretin and Gut Hormone Expression in Human Alcohol-Related Brain Damage: Opportunities for Therapeutic Targeting

**DOI:** 10.3390/biom16010099

**Published:** 2026-01-07

**Authors:** Suzanne M. de la Monte, Ming Tong, Rolf I. Carlson, Greg Sutherland

**Affiliations:** 1 Departments of Pathology and Laboratory Medicine, Neurology, Neurosurgery and Medicine, Rhode Island Hospital, Alpert Medical School of Brown University, Brown University Health, Providence, RI 02903, USA; 2 Department of Medicine, Rhode Island Hospital, Alpert Medical School of Brown University, Brown University Health, Providence, RI 02903, USA; 3 Charles Perkins Centre and School of Medical Sciences, Faculty of Medicine and Health, The University of Sydney, Camperdown, NSW 2006, Australia

**Keywords:** alcohol use disorder, incretin, neurodegeneration, alcohol-related brain damage, human, postmortem brain, gut hormones

## Abstract

Background: Alcohol use disorder (AUD) is associated with chronic heavy or repeated binge alcohol abuse, which can cause alcohol-related brain damage (ARBD) marked by neurobehavioral, cognitive, and motor deficits. The anterior frontal lobe and cerebellar vermis are two of the major targets of ARBD in humans with AUD and in experimental alcohol exposed models. Alcohol’s neurotoxic and neurodegenerative effects include impairments in signaling through insulin and insulin-like growth factor (IGF) pathways that regulate energy metabolism. This human AUD study was inspired by a recent report suggesting that dysfunction of the frontal lobe incretin network in experimental ARBD is linked to known impairments in brain insulin/IGF signaling. Objective: The overarching goal was to investigate whether AUD is associated with dysfunction of the brain’s incretin network, focusing on the cerebellum and frontal lobe. Methods: Fresh frozen postmortem cerebellar vermis and anterior frontal lobe tissues from adult male AUD (*n* = 6) and control (*n* = 6) donors were processed for protein extraction. Duplex enzyme-linked immunosorbent assays (ELISAs) were used to assess immunoreactivity to neurofilament light chain (NfL) as a marker of neurodegeneration. A multiplex ELISA was used to measure immunoreactivity to a panel of gut hormones, including incretin polypeptides. Results: AUD was associated with significantly increased NfL immunoreactivity in both the cerebellar vermis and anterior frontal lobe. However, the patterns of AUD-related alterations in gut hormone immunoreactivity differed regionally. AUD reduced pancreatic polypeptide immunoreactivity in the cerebellar vermis, and GIP, GLP-1, leptin, and ghrelin in the frontal lobe. Conclusions: (1) Increased NfL may serve as a useful biomarker of neurodegeneration in AUD. (2) AUD’s adverse effects on neuroendocrine signaling networks differ in the cerebellar vermis and anterior frontal region, although both are significant targets of ARBD. (3) The finding of AUD-associated reductions in frontal lobe GIP and GLP-1 suggests that therapeutic targeting with incretin receptor agonists may help restore energy metabolism and neurobehavioral and cognitive functions linked to their networks.

## 1. Introduction

Alcohol use disorder (AUD), marked by binge, chronic, or both modes of excessive alcohol consumption, increases the risk for developing alcohol-related brain damage (ARBD). ARBD encompasses a spectrum of neuropathologic abnormalities caused by neurotoxic and degenerative effects of alcohol [1,2,3,4,5]. The clinical manifestations of AUD include neurobehavioral, cognitive, and motor dysfunctions. Brain atrophy in AUD and ARBD is most pronounced in the cerebral cortex, diencephalon (including hypothalamus), white matter, cerebellum, hippocampi, and subcortical nuclei. AUD-and ARBD-related white matter pathology is marked by dysfunction and loss of oligodendrocytes, leading to demyelination, dysmyelination, and ultimately axonal degeneration. The attendant compromise of neuronal conductivity and synaptic integrity [1,2], contributes to progressive and sustained cognitive-motor deficits [3,4,5]. Despite well-documented patterns of neurodegeneration in AUD and ARBD, unlike many brain diseases linked to cognitive-behavioral dysfunctions including Alzheimer’s disease (AD), Parkinson’s disease (PD), and frontotemporal lobar degeneration (FTLD), there are no known diagnostic features or biomarkers of ARBD, apart from Wernicke’s encephalopathy, which is largely due to the combined effects of alcohol neurotoxicity and thiamine/nutritional-deficiency. Cerebellar cortex degeneration is another characteristic feature of AUD/ARBD [6], but not AD, PD, or FTLD. White matter and diencephalic (including hypothalamus) atrophy are notable signature pathologies in AUD [3], but they overlap with neuropathological changes in other neurodegenerative diseases including AD, PD and FTLD [7,8]. Data from various epidemiological, neuropsychological, and neuroimaging studies suggest that AUD increases brain aging and susceptibility to specific forms of neurodegeneration, such as AD, whose dominant risk factors are aging [9], yet postmortem and experimental animal model studies have not demonstrated independent and distinct characteristic AD pathology following heavy alcohol exposure alone. These knowledge gaps justify further research to better understand the specific brain pathologies in AUD and to identify potential biomarkers for early-stage detection and monitoring of related neurodegeneration.

Information about underlying mediators of brain atrophy and neurodegeneration in AUD and experimental ARBD stemmed from studies demonstrating adverse effects of chronic, high-level ethanol exposure on the integrity of insulin and insulin-like growth factor (IGF) signaling networks in the brain [10]. It is noteworthy that insulin and IGF receptors are abundantly expressed in brain regions that are known to be major targets of AUD [11,12]. Experiments conducted over the past several decades demonstrated that disruption of the insulin/IGF networks at various levels within the signaling cascades compromises energy metabolism and homeostasis, which are needed for cell survival, neuronal plasticity, and white matter integrity. The short-term adverse effects of ethanol exposure are often mediated by the combined effects of inhibiting phosphorylation of intracellular signaling, oxidative injury, neuroinflammation, and cell death. In contrast, the underlying drivers of ethanol’s long-term adverse effects remain incompletely understood. However, new opportunities appear to have emerged with the growing appreciation of the broad critical roles of incretin signaling in maintaining metabolic homeostasis and insulin network functions throughout the body, including the brain [13,14,15]. These concepts led to the hypothesis that the chronic adverse effects of ethanol may be mediated by integrin-related network inhibition.

The incretin family of hormones interacts with receptors throughout the body, including in the central nervous system (CNS). Incretin mRNAs encoding glucagon-like peptide 1 (GLP-1) and glucose-dependent insulinotropic polypeptide (GIP) have been localized to various brain regions [16]. GLP-1, now the most studied of the incretins, stimulates glucose-dependent insulin secretion and insulin biosynthesis, promotes healthy insulin signaling, regulates blood glucose, inhibits glucagon secretion and gastric emptying, and curtails food intake. GLP-1 receptor agonists (RAs) and GIP-RAs are of particular interest because strong preclinical and clinical data show that, beyond their insulin-stimulating effects to achieve glycemic control, these drugs have pleiotropic CNS actions and positively impact neuronal functions such as energy homeostasis, neurogenesis, and neuroprotection from cognitive decline [17]. Mechanistically, incretins decrease neuroinflammation, oxidative stress, dysregulated metabolism, and impairments in plasticity and cell survival [17]. Given that GLP-1R is expressed on all CNS cell types (neurons, oligodendroglia, astrocytes, microglia, endothelial cells, and pericytes) [18], and insulin/IGF signaling is critical for maintaining white matter integrity, which is consistently targeted in ARBD, it is of interest to explore the potential role of ethanol-impaired incretin signaling in relation to AUD. Correspondingly, in a recent study, significant impairments in frontal lobe white matter expression of incretins were observed in an experimental model of ARBD, in which insulin/IGF signaling networks had previously been shown to be inhibited [19]. The present study was designed to determine if AUD in humans was also associated with frontal lobe impairments in incretin-network molecule expression. In addition, the efforts were extended to assessing whether responses in the cerebellar vermis, another important target of AUD neurodegeneration, mirrored or diverged from those observed in the frontal lobe.

Although data on the effects of incretin receptor agonists on oligodendrocytes, myelin, or white matter are scant, there is some evidence that long-acting incretin RAs can support Schwann cell survival and myelination in insulin-resistant states [20]. In addition, GLP-1 RA treatments can support axonal regeneration and remyelination, as shown in models of multiple sclerosis [21,22], and can improve functional recovery after spinal cord injury [23], enhance the survival of mature Olig2+/CC1+ oligodendrocytes, and promote remyelination after cuprizone-induced neuropathy [24]. Whether incretin receptor agonist treatments could prevent or reduce the severity of ARBD is unknown. Apart from their potential benefits related to the structural pathology of ARBD, clinical trials have been launched to address AUD-related neurobehavioral problems, such as craving and addiction, which lead to ARBD [13,25,26,27,28]. The long-term outcomes of those investigations are still pending. Beyond incretins/incretin receptor agonists are the incretin-related molecules such as amylin, which is co-secreted with insulin and has neuroprotective and pro-metabolic effects in the brain, and oxyntomodulin, which acts as a dual agonist for GIP and GLP-1, and exerts neuroprotective, neurotrophic, and pro-metabolic effects in the brain [29]. Interest in these molecules has grown from the realization that targeting metabolic pathways to restore brain function will likely require multi-pronged approaches.

## 2. Materials and Methods

### 2.1. Human Subjects

Human postmortem brain tissue samples were obtained from donors with a clinical diagnosis of AUD or from controls with no history of alcohol or other substance use disorder. The brains were banked at the New South Wales Brain Tissue Resource Centre (NSW BTRC) in Sydney, Australia. The NSW BTRC and its associated donor program have ethics approval from the NSW Government Health authority/University of Sydney to bank postmortem brains from deceased subjects with documented histories of alcohol abuse, or normal, non-substance abusing control (ref# X11-0107&HREC/11/RPAH/147). The NSW BTRC has ethics approval that permits any adult aged 18 years or older to consent to brain donation. The cases analyzed were between 40 and 70 years of age. All donors provided written informed consent to participate in this study. The samples were de-identified prior to transfer from the BTRC to Brown University Health. A Tissue Transfer Agreement outlining the conditions of tissue usage was required to be completed prior to making the tissue samples available. were obtained from the New South Wales Brain Tissue Research Centre (BTRC). The human tissue research was approved by the BTRC Scientific Advisory Committee, the University of Sydney Human Research Ethics Committee (2018/HE000477), and the Brown University Health Institutional Review Board (CMTT/PROJ:#013024; approved 5 June 2017).

### 2.2. Human Brain Tissue Homogenization for Protein Studies

Fresh frozen tissue cores (6 mm diameter) from the anterior frontal lobe and cerebellar vermis were stored at −80 °C. Two aliquots per region/case were homogenized in 5 volumes of weak lysis buffer (50 mM Tris (pH 7.5), 150 mM NaCl, 5 mM EDTA (pH 8.0), 50 mM NaF, and 0.1% Triton X-100) containing protease inhibitors (1 mM PMSF, 0.1 mM TPCK, 2 µg/mL aprotinin, 2 µg/mL pepstatin A, and 1 µg/mL leupeptin) and 10 mM Na_3_VO_4_ to inhibit phosphatases. The samples were homogenized using a TissueLyser II instrument (Qiagen, Germantown, MD, USA) with 5 mm-diameter stainless steel beads. The clarified supernatants from centrifuging the samples at 14,000 rpm for 10 min at 4 °C were stored at −80 °C. Protein concentrations were determined using the bicinchoninic acid (BCA) assay. The sources of reagents and instruments used for this research are listed in Supplementary (Table S1).

### 2.3. Duplex Enzyme-Linked Immunosorbent Assays (ELISAs)

Duplex ELISAs were used to measure neurofilament light chain (NfL) immunoreactivity with results normalized to large acidic ribosomal protein (RPLPO) as the loading control [30,31,32]. The assays were performed in triplicate with 50 ng protein samples in 50 µL bicarbonate binding buffer added to 96-well MaxiSorp plates. After overnight adsorption at 4 °C, non-specific binding sites were masked with Superblock TBS. The samples were incubated overnight at 4 °C with rabbit polyclonal anti-NfL (0.6 µg/mL; Research Resource Identifier (RRID) #12998-1-AP; Abcam, Boston, MA, USA). Immunoreactivity was detected with horseradish peroxidase (HRP)-conjugated secondary antibodies and the Amplex UltraRed soluble fluorophore. Fluorescence intensity was measured (Ex 530 nm/Em 590 nm) in a Spectra-Max M5 Multimode Plate Reader (Molecular Devices, Sunnyvale, CA, USA). After rinsing the reactions in Tris-buffered saline (TBS), the samples were incubated with biotin-conjugated mouse monoclonal anti-RPLPO (0.1 µg/mL; RRID# ab10738968; Santa Cruz, Dallas, TX, USA), followed by streptavidin-conjugated alkaline phosphatase. Immunoreactivity was detected with 4-Methylumbelliferyl phosphate (4-MUP) (Ex 360 nm/Em 450 nm). Fluorescence was measured in a SpectraMax M5(Molecular Devices, San Jose, CA, USA). The calculated ratios of target protein to RPLPO were used for statistical comparisons.

### 2.4. Multiplex ELISA

A human Multiplex (7-Plex) Gut Hormone magnetic bead-based panel (Millipore #HGT-68K; Burlington, MA, USA) was used to measure ghrelin, leptin, glucose-dependent insulinotropic polypeptide (GIP), glucagon-like peptide 1 (GLP-1), pancreatic polypeptide (PP), Peptide YY/Neuropeptide Y (PYY), and insulin in brain tissue. The proteins, their abbreviations, gene names, functions, and alcohol effects are listed in Table S2. The assay was performed according to the manufacturer’s protocol. In brief, after incubating samples containing 250 µg of protein with antibody-conjugated magnetic beads, immunoreactivity was detected using biotinylated secondary antibodies and Streptavidin-conjugated phycoerythrin. Immunoreactivity was measured in a MagPix Instrument (Diasorin, Austin, TX, USA), and the results were analyzed using xPONENT software(Version 4.3). Standard curves for each analyte were included in all assays.

### 2.5. Data Analysis

GraphPad Prism 10.5 software (GraphPad Software Inc., Boston, MA, USA) was used to analyze data and generate graphs. Prior to making statistical comparisons, GraphPad data analysis tools were used to ensure that the assumptions for normality were met. Violin plots depict the distribution of results, including the median (mid-horizontal bar), the bottom (lower horizontal line) and top (upper horizontal line) quartiles, and the range (tips). Welch *t*-tests were used to compare clinical and relevant postmortem factors in the control and AUD cases. A two-way analysis of Variance (ANOVA) with post hoc Tukey–Kramer multiple comparisons was used to evaluate the effects of AUD on immunoreactivity across brain regions. The analyses included corrections for multiple comparisons (false discovery rate of 5%). Heatmaps were used to summarize relative differences in gut hormone expression within and between the AUD and control groups. Software-generated statistically significant (*p* ≤ 0.05) differences are displayed within the graph panels. The “trend-wise” *p*-values (0.05 < *p* < 0.10) are noted only as potentially bordering on significance. However, they are nonsignificant [33] and therefore must be regarded with caution.

## 3. Results

### 3.1. Brain Donor Characteristics

**Figure 1:**
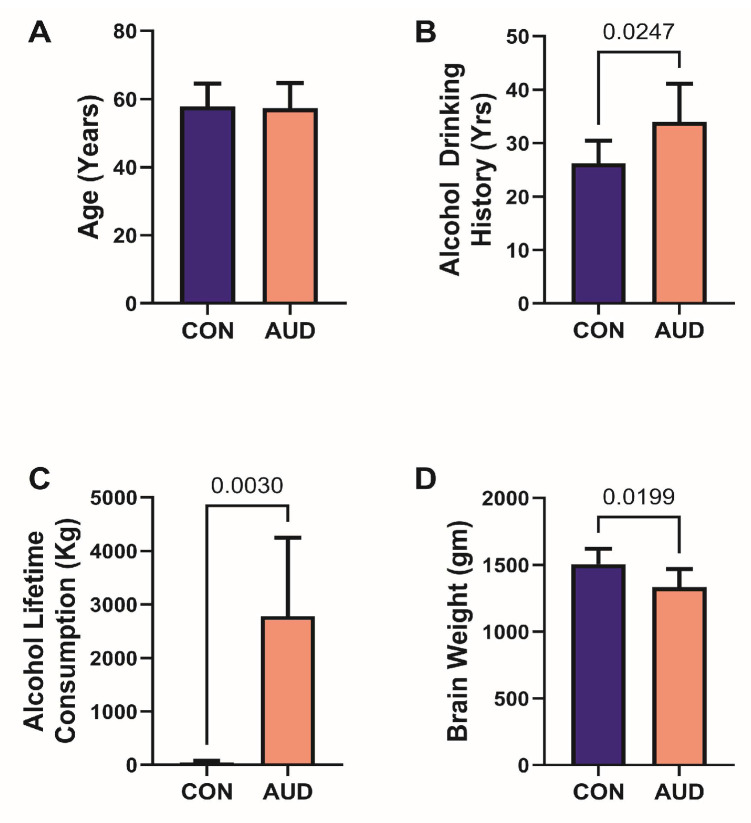
Human subject brain donors. Comparisons between the control (Con) and AUD groups (*n* = 6 cases per group) revealed (**A**) similar mean ages, and significantly (**B**) longer histories of alcohol drinking, (**C**) greater lifetime consumption of alcohol, and (**D**) lower mean brain weight in the AUD group. The graphs display the mean ± S.D. of results. Inter-group comparisons were made by Welch *t*-test. Significant *p*-values are displayed. See Table 1 for additional information.

This study included 12 human donor subjects; 6 had a clinical diagnosis of AUD, and 6 were controls (Table 1 and Figure 1).

All subjects were male. The mean ages (Figure 1A) and age ranges (50–69 or 50–70) were similar for the two groups. Corresponding with their diagnoses, the mean alcohol drinking history duration (years) (Figure 1B) was significantly longer, the mean lifetime alcohol consumption (kg) (Figure 1C) was significantly greater, and the mean brain weight was lower (Figure 1D) in the AUD group. The frequencies of chronic smoking were similar (Table 1). Other factors reflecting tissue integrity and therefore potentially affecting data quality, including postmortem interval, RNA integrity number, and brain tissue pH, were similar for the control and AUD groups (Table 1).

### 3.2. AUD Marker of Neurodegeneration

**Figure 2:**
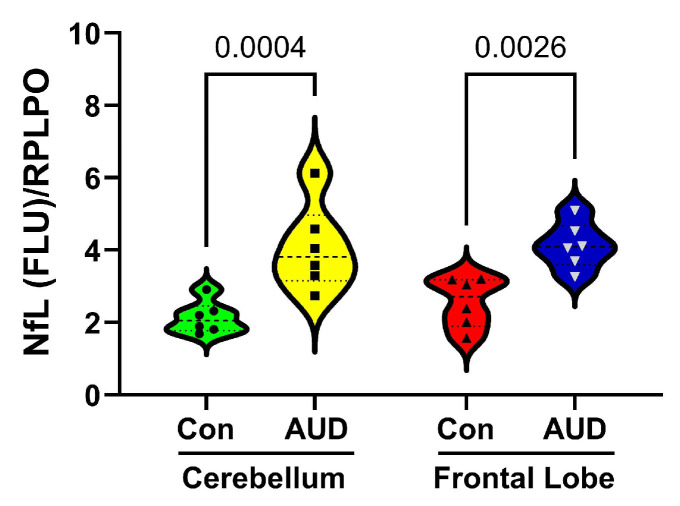
Postmortem cerebellar vermis and frontal lobe brain tissue samples from human control (Con; *n* = 6) and AUD (*n* = 6) donors were analyzed for neurofilament light chain (NfL) immunoreactivity by duplex ELISA with results normalized to RPLPO. Violin plots display inter-group differences in the levels of immunoreactivity by brain region. Two-way ANOVA with the post hoc tests revealed significantly higher levels of NfL in the AUD compared with Con cerebellar and frontal lobe samples. The software-calculated *p*-values are shown.

A duplex ELISA measured neurofilament light chain (NfL) immunoreactivity as an index of neurodegeneration [34,35]. Results were normalized to RPLPO as a loading control. Two-way ANOVA detected significant effects of subject group/diagnosis (F(1,20) = 25.59, *p* < 0.0001), but not brain region or diagnosis x brain region interactions. The post hoc Tukey–Kramer test comparing within-brain region effects revealed significantly higher AUD levels of NfL in both the cerebellar and frontal lobe samples (Figure 2).

### 3.3. Gut Hormone 7-Plex ELISA

**Figure 3:**
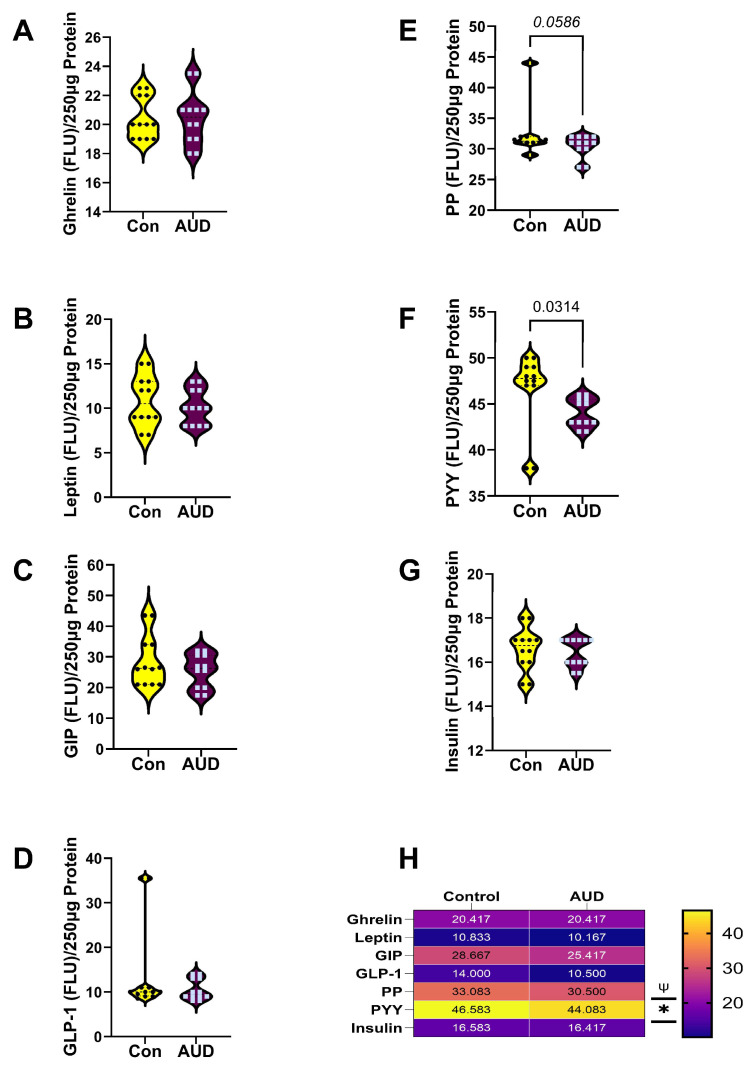
Effects of AUD on cerebellar vermis expression of gut hormone polypeptides. Immunoreactivity to (**A**) Ghrelin, (**B**) Leptin, (**C**) GIP, (**D**) GLP-1, (**E**) PP, (**F**) PYY, and (**G**) Insulin was measured in human control (Con) and AUD cerebellar vermis homogenates using a 7-plex magnetic bead-based panel. Immunoreactivity (fluorescent light units; FLU) was quantified in 250 µg protein samples. Purified commercial standards were included in all assays to calculate levels of immunoreactivity. Inter-group comparisons were made by two-way ANOVA (Table 2) and the post hoc Tukey–Kramer test (for within-row comparisons). The false discovery rate was set at 5%. *p* ≤ 0.05 was considered significant. 0.05 < *p* < 0.10 represents a statistical trend (italics font). (**H**) Heatmap summary of AUD’s effects on incretin and gut hormone polypeptide immunoreactivity. The color scales correspond to mean immunoreactivity levels shown within the tiles (* *p* < 0.05; ψ 0.05 < *p* < 0.10).

**Figure 4:**
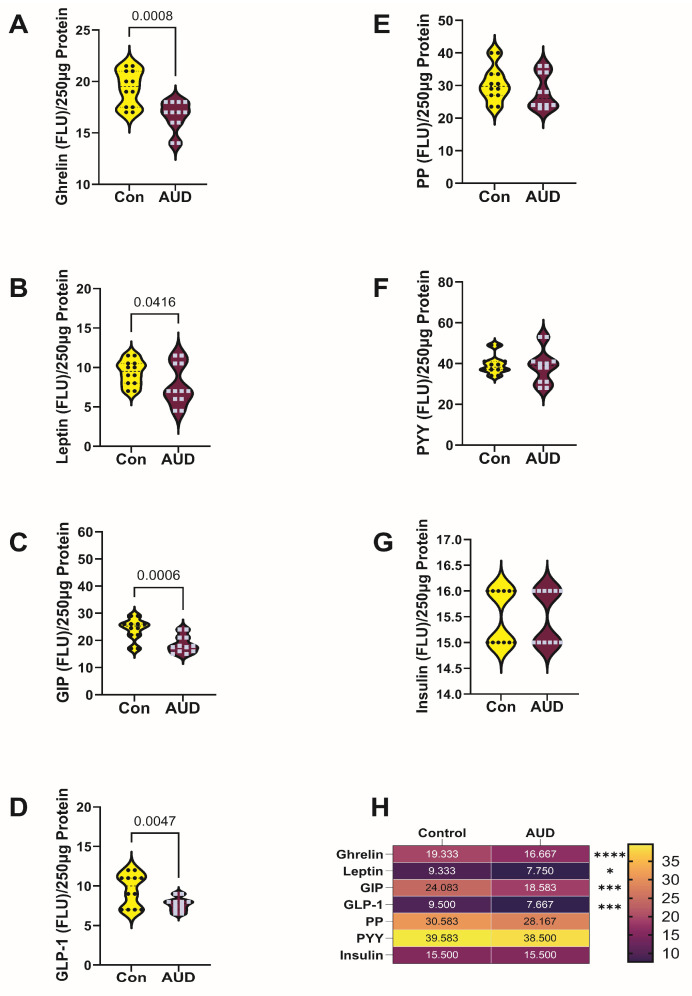
Effects of AUD on frontal lobe expression of gut hormone polypeptides. Immunoreactivity to (**A**) Ghrelin, (**B**) Leptin, (**C**) GIP, (**D**) GLP-1, (**E**) PP, (**F**) PYY, and (**G**) Insulin was measured in frontal lobe tissue homogenates from 6 control (Con) and 6 AUD donors using a 7-plex magnetic bead-based panel. Immunoreactivity (fluorescent light units; FLU) was quantified in 250 µg protein samples. Purified commercial standards were included in all assays to calculate levels of immunoreactivity. Inter-group comparisons were made by two-way ANOVA (Table 2) and the post hoc Tukey–Kramer test (for within-row comparisons). The false discovery rate was set at 5%. *p* ≤ 0.05 was considered significant. (**H**) The heatmap generated with GraphPad Prism 10.5, summarizes AUD’s effects on incretin and gut hormone polypeptide immunoreactivity. The color scales correspond to mean immunoreactivity levels shown within the tiles (**** *p* < 0.0001; *** *p* < 0.001; * *p* < 0.05). Dots/squares reflect individual values within the violin plots.

The results obtained with the 7-plex Gut Hormone ELISA panel were analyzed by two-way ANOVA, which demonstrated significant effects of AUD and biomarker type, but no interactive effects of AUD × biomarker type in either the cerebellum or frontal lobe samples (Table 2). Graph panels depict the effects of AUD on gut hormone immunoreactivity in cerebellar (Figure 3) and frontal lobe (Figure 4) samples, along with the results of ANOVA post hoc within-row comparison tests (False discovery rate = 0.05). In addition, heatmaps summarize the comparative effects of AUD on gut hormone expression by brain region.

For the cerebellar vermis, the mean levels of ghrelin (Figure 3A), leptin (Figure 3B), GIP (Figure 3C), GLP-1 (Figure 3D), and insulin (Figure 3G) were similar in the control and AUD samples. In contrast, AUD was associated with a significant reduction in PYY (Figure 3F) and a statistical trendwise reduction in PP (Figure 3E). Concerning the anterior frontal lobe, post hoc tests demonstrated significant AUD-associated reductions in ghrelin (Figure 4A), leptin (Figure 4B), GIP (Figure 4C), and GLP-1 (Figure 4D), but similar mean levels of PP (Figure 4E), PYY (Figure 4F), and insulin (Figure 4G) relative to control.

Heatmaps summarized the brain regional effects AUD on gut hormone/incretin polypeptide immunoreactivity (Figure 3H and Figure 4H). Of note, in the cerebellum, although there were no significant between-group differences in GIP and GLP-1 immunoreactivity, the mean levels of both incretins were reduced by AUD, paralleling the responses in the frontal lobe.

## 4. Discussion

This study investigated the effects of AUD on cerebellar and frontal lobe expression of incretins and related gut hormone polypeptides and was designed, in part, to validate recent findings in an experimental model of ARBD [19]. The underlying premise is that chronic heavy alcohol consumption constitutively impairs brain insulin/IGF signaling, which is needed to support crucial functions such as energy metabolism, neuronal plasticity, cell survival, myelin integrity, cognition, and motor coordination. Previous studies in experimental models have shown that ethanol inhibits insulin/IGF signaling through insulin receptor substrate (IRS) and downstream through phosphatidylinositol-3-kinase (PI3K), Akt, and the mechanistic target of rapamycin (mTOR) [36,37]. Major brain structural targets of ethanol and associated impairments in insulin/IGF signaling include the frontal lobe, temporal lobe, white matter, and cerebellum [36]. Notable neuropathological findings include white matter atrophy with myelin loss, followed by axonal loss, neuronal loss in the cerebellar cortex, and atrophy with gliosis in the cerebral cortex [36,38]. Experimental models correlated ethanol-mediated brain structural pathologies with impairments in insulin/IGF signaling networks, and neurobehavioral dysfunction [39]. The aggregate findings raised the question of whether the broad adverse effects of ethanol were mediated by a more proximal “lesion” that could cast a wide net and negatively affect multiple pathways and systems simultaneously.

The potential role of impaired integrin-related network signaling as the driver of dysregulated insulin/IGF signaling in AUD/ARBD was suggested by the overlap among brain structures that are prominently targeted by the neurotoxic effects of ethanol and the abundant localization of insulin/IGF receptors and incretin/gut hormone polypeptide expression, i.e., frontal and temporal lobes, hypothalamus, hippocampus, and cerebellum [36,40]. This realization led to the analysis of an experimental model of ARBD, which demonstrated that chronic heavy ethanol exposure significantly inhibited frontal lobe expression of GIP, amylin, ghrelin, leptin, C-peptide, and glucagon [19]. Since the findings suggest that treatment with incretin receptor agonists could restore or enhance downstream signaling via insulin/IGF, it was important to assess their translational relevance and determine whether similar abnormalities exist in human AUD brains. Moreover, although both the frontal lobes and cerebellum utilize insulin/IGF signaling networks that are impaired by ethanol, it was unknown whether the adverse effects of AUD on incretin-related networks would be similar or disparate across brain regions, a phenomenon with clinical significance.

The human postmortem tissue samples were from well-characterized participants who donated their brains for research. Importantly, both cerebellar and frontal lobe AUD specimens showed significant increases in NfL immunoreactivity. Previous studies linked elevated levels of NfL immunoreactivity to various neurodegenerative diseases [34,35,41,42,43]. Therefore, although not specific, the increased NfL observed in AUD brains could serve as a general biomarker of neurodegeneration linked to AUD/ARBD. This point is worthy of consideration because, apart from white matter atrophy, gray and white matter gliosis, and neuronal loss, all of which worsen with longer duration and larger lifetime volume consumption of alcohol [44], there are no readily measured or monitored pathologies that are diagnostic of ARBD. Future studies should assess the utility of measuring serum NfL in individuals with AUD to determine the extent to which elevated levels correlate with cognitive-motor impairments and brain atrophy.

Further analysis of gut hormone and incretin expression revealed distinct regional differences in the effects of AUD. In the cerebellum, the abnormalities were restricted to PP and PYY, whereas in the frontal lobe, multiple significant abnormalities in incretin and related polypeptide expression were observed. The reduced PP and PYY immunoreactivities in AUD cerebellum contrast with previous findings that ethanol exposure had no significant effects on serum levels of PYY or PP [45]. However, ethanol-related cerebellar abnormalities may not be detected in serum-based assays.

The cerebellar vermis, a target of ARBD [46], plays a dominant role in mediating motor functions such as balance, posture, and coordinated locomotion, but it also contributes positively to motivation, reward learning, and social/emotional behaviors [47]. Pancreatic polypeptide and peptide YY are members of a family of pancreatic polypeptides that include Neuropeptide Y (NPY) [48]. The cerebellum is one of the many brain regions in which PP and PYY are expressed. NPY-related genes expressed throughout the brain function in learning and memory via hippocampal circuitry. In addition, NYPs regulate stress responses, blood pressure, heart rate, metabolism, and immune functions [49]. The relatively high levels of NPY, PP, and PYY expressed during development correspond to their roles in neuronal growth, plasticity, and anti-inflammatory functions. The finding of reduced cerebellar PYY and PP expression in AUD is novel and may reflect non-motor, i.e., cognitive deficits in cerebellar function. Additional studies are needed to link these tissue responses with other clinical or pathological features of AUD and ARBD.

In the frontal lobe, the AUD-associated reductions in ghrelin and leptin are significant because ghrelin inhibition likely corresponds to deficits in neuronal plasticity required for memory and learning, and reduced leptin reflects impaired energy balance and expenditure. These human brain tissue findings correspond with previous observations in chronic experimental ethanol feeding models that lead to ARBD [19,50], and humans with Alzheimer’s neurodegeneration [51]. Besides regulating food intake and body weight, ghrelin and leptin have important roles in synaptic plasticity utilized in response to changes in energy status and metabolism [52,53]. Therefore, their reductions in AUD could reflect declines in synaptic plasticity. The delicate balance of restoring these functions while dampening craving is an important therapeutic consideration, since further reductions in ghrelin and leptin could adversely affect cognitive behavior and brain metabolic function.

Evidence suggests that incretin secretion is modulated by circadian rhythm [54,55] and that dysregulation of circadian clock genes is a feature of neurodegenerative diseases, including Alzheimer’s, and is associated with altered signaling through Akt/mTOR pathways [56]. Of particular relevance to the present study is that alcohol ingestion also disrupts circadian rhythms in various organs and tissues, including the brain [57] and skeletal muscle [58], and it impairs nocturnal and diurnal leptin secretion [59]. Therefore, alcohol-mediated disruption of circadian rhythm could mechanistically account for AUD’s inhibitory effects on neuroendocrine, including incretin polypeptide expression in the brain. Unfortunately, since circadian rhythm-clock gene expression profiles were not investigated in this study, the potential impact of AUD-mediated circadian rhythm disruption in relation to the observed inhibitory effects on frontal lobe neuroendocrine, including incretin expression, is unknown. However, future studies should incorporate related analyses to better elucidate the pathogenic factors underlying AUD-associated dysregulation of neurometabolic signaling in the brain.

The AUD frontal lobe samples had significantly reduced levels of both the GLP-1 and GIP, which partially correspond with recent experimental ARBD findings [19], but differ from the unaltered incretin expression observed herein in the cerebellar vermis. These results underscore the importance of sampling different brain regions and recognizing that the mediators of neurodegeneration may differ across them. At the same time, this study provides evidence that AUD is associated with reduced incretin expression in the brain. Moreover, in the experimental animal model study, alcohol-related impairments in brain incretin expression were detected in exosomes isolated from serum [19], suggesting opportunities for non-invasive detection and monitoring of disease. Although this study was observational rather than treatment-based, the findings nonetheless support the hypothesis that treatment with long-acting incretin receptor agonists targeting both GLP-1 and GIP receptors may benefit individuals since remediation of both deficiencies will likely be needed to support brain metabolic functions. Dual treatment with GLP-1 and GIP receptor agonists is feasible because relevant hybrid pharmacological compounds already exist and are used clinically to treat insulin resistance diseases, including diabetes mellitus, obesity, and a growing list of related pathologies [13,25,26]. Regarding ARBD, preclinical studies have demonstrated significant benefits of incretin receptor agonist treatment for reducing alcohol intake [13,25,26,27,28,29]. Multiple clinical studies and formal trials to address craving and addiction behaviors in AUD have been launched [60,61,62], but the long-term outcomes are still pending.

## 5. Conclusions

AUD was associated with increased NfL expression, a marker of neurodegeneration. Additional research is needed to determine whether a serum- or cerebrospinal fluid-based assays of NfL immunoreactivity can provide an independent biomarker of AUD in the clinical setting. The AUD-associated reductions in frontal lobe GIP and GLP are significant because the frontal lobes are major targets of ARBD, and given their roles in regulating energy metabolism linked to insulin networks, the findings support the concept that incretin-receptor agonist treatments could be used to remediate cognitive-behavioral pathologies in AUD. Finally, additional abnormalities in ghrelin, leptin, PP, and PYY, differentially detected in the frontal lobe or the cerebellar vermis, indicate that future therapeutic approaches must address brain-region-specific profiles of neuroendocrine network dysregulation in AUD.

## 6. Limitations of This Study

Conclusions drawn from this study are cautioned due to the modest subject group sizes and the inclusion of only males. Despite the advantages of studying well-characterized AUD subjects who were heavy drinkers and lacked complicated additional substance abuse histories, the population may not be fully representative of most AUD worldwide. Furthermore, although difficult to obtain, this type of research could be strengthened by including participants with prior heavy alcohol abuse histories who subsequently ceased drinking, to better understand the reversibility of neurodegeneration and dysregulated incretin/neuroendocrine networks.

## Figures and Tables

**Table 1:** Human Subject Brain Donors.

Characteristics	Controls	AUD	*p*-Value
# Cases (all Male)	6	6	
Age Range (Years)	50–69	50–70	
Smoking History (Y/N)	3/6	4/6	N.S.
Postmortem Interval (h)	22.17 ± 6.43	32.17 ± 24.81	N.S.
RNA Integrity Number	7.50 ± 0.70	6.84 ± 1.35	N.S.
Brain pH	6.61 ± 0.21	6.66 ± 0.23	N.S.

Characteristics of control and AUD deceased donors. The number (#) of cases and smoking history values reflect the number or proportion of cases. Postmortem interval, RNA integrity index, and brain pH values reflect the mean ± S.D. Intergroup comparisons of the mean values were made using Welch *t*-tests. Proportional differences in smoking history were compared using Chi-square tests. N.S. = not statistically significant. See Figure 1 for inter-group comparisons of the mean age, alcohol consumption history durations, lifetime quantity of alcohol consumed, and brain weight.

**Table 2:** AUD Brain Tissue Multiplex ELISAs: Two-way ANOVA Results.

Brain Region	AUD-Factor F-Ratio	*p*-Value	Biomarker F-Ratio	*p*-Value	AUD × Biomarker F-Ratio	*p*-Value
Cerebellum	**7.035**	**0.0088**	**186.6**	**<0.0001**	0.6798	N.S.
Frontal Lobe	**13.76**	**0.0003**	**208.3**	**<0.0001**	1.252	N.S.

Human postmortem cerebellar vermis and anterior frontal lobe tissue samples from control and AUD participants were analyzed with a 7-plex ELISA panel. The data were analyzed by two-way ANOVA to compare the effects of AUD, biomarkers, and AUD × Biomarker interactions. The calculated F-ratios and *p*-values are indicated. Significant effects (*p* ≤ 0.05) are highlighted with bold font. N.S. = not significant. The results of post hoc multiple comparisons tests are shown in Figure 3 and Figure 4.

## Data Availability

The datasets created and/or analyzed in this study are available from the corresponding author upon reasonable request.

## Supplementary Materials

The following supporting information can be downloaded at https://www.mdpi.com/article/10.3390/biom16010099/s1: Table S1: Reagents and Instrument Sources; Table S2: Human 7-Plex Gut/Metabolic Hormone Pane [63,64,65,66,67,68,69,70,71,72,73,74,75].

## Abbreviations

The following abbreviations are used in this manuscript:

AUD	Alcohol Use Disorder
ARBD	Alcohol-related brain damage
ELISA	Enzyme-linked immunosorbent assay
NfL	Neurofilament light chain
GIP	Glucose-dependent insulinotropic polypeptide
GLP-1	Glucagon-like peptide-1
IGF-1	Insulin-like growth factor-1
CNS	Central nervous system
RA	Receptor agonist
NSW	New South Wales
BTRC	Brain tissue resource center
PP	Pancreatic polypeptide
PYY	Peptide YY/Neuropeptide Y
ANOVA	Analysis of variance
RPLPO	Large acidic ribosomal protein
