# Supplementary material for: Impaired Brain Incretin and Gut Hormone Expression in Human Alcohol-Related Brain Damage: Opportunities for Therapeutic Targeting
Source: Biomolecules. 2026 Jan 7;16(1):99. doi: 10.3390/biom16010099 (PMC12838672; doi:10.3390/biom16010099)
Supplement: Supplementary file 1 [file biomolecules-16-00099-s001.zip › biomolecules-3997999-supplementary.pdf]

Impaired Brain Incretin and Gut Hormone Expression in Human Alcohol-Related Brain Damage: Opportunities for Therapeutic Targeting

Suzanne M. de la Monte, Ming Tong, Rolf I. Carlson, and Greg Sutherland

Table S1. Reagents and Instrument Sources

**Reagents**	**Commercial Source**
Bicinchoninic acid reagents, secondary HRP-conjugated antibodies, Superblock-TBS, MaxiSorp 96-well plates	ThermoFisher Scientific, Bedford MA USA
Amplex UltraRed soluble fluorophore, 4-Methylumbelliferyl phosphate (4-MUP)	Invitrogen, ThermoFisher, Bedford MA, USA
Alkaline phosphatase streptavidin, Proton Biotin Protein Labeling Kit	Vector Laboratories Inc, Newark CA USA
Miscellaneous fine chemical reagents	CalBiochem/Millipore Sigma, Burlington, MA, USA; Pierce Chemical, Dallas, TX, USA; or Sigma-Aldrich Co., St. Louis, MO, USA
**Multiplex Panels**	
7-Plex Human Gut Hormone Panel; Millipore #HGT-68K	Millipore, Burlington, MA, USA
**Instruments**	
Luminex MAGPIX	Diasorin, Austin TX, USA
SpectraMax M5 Multimode Microplate reader	Molecular Devices, San Jose, CA USA
TissueLyser II	The Cavey Laboratory Engineering Co., Guildford, Surrey, UK

Table S2. Human 7-Plex Gut/Metabolic Hormone Pane

Factor	Gene Name	Functions	Alcohol Effects
**Ghrelin**	*GHRL*; Ghrelin and Obestatin Prepropeptide	Ligand for growth hormone secretagogue receptor type 1; induces growth hormone release from the pituitary; regulates growth; stimulates appetite; induces adiposity; stimulates gastric acid secretion. Regulates synaptic function and plasticity related to feeding behavior, as well as cognition [52]; inhibited in AD neurodegeneration [51]; promotes dendritic spin remodeling in hippocampal neurons [53].	Inhibits ghrelin in serum [63]; increases alcohol craving in humans [64]; GLP1-receptor agonists reduce alcohol intake in experimental models [25]; experimental in vivo model of ARBD reduced ghrelin expression in the frontal lobe [50]; alcohol-related reduction in frontal lobe ghrelin expression was associated with reduced ghrelin immunoreactivity in serum exosomes [19].
**GIP**	*GIP* Glucose-dependent insulinotropic polypeptide; Gastric Inhibitory Polypeptide	Potent stimulator of insulin secretion, maintains glucose homeostasis; stimulates lipoprotein lipase; modulates fatty acid metabolism; poor inhibitor of gastric acid secretion; promotes satiety leading to weight loss [65]. Expressed in human and rodent hypothalamus [16]. Enhances brain neuronal plasticity and neuroprotection [13].	Receptor agonists reduce alcohol consumption in obese people [61]. Inhibited in the frontal lobe by chronic alcohol feeding in an experimental model [19].
**GLP-1**	*GCG;* Glucagon-like peptide 1	Potent stimulator of glucose-dependent insulin release, stimulates glucose disposal, and promotes satiety [66]; suppresses plasma glucagon; modulates gastric motility; promotes growth of intestinal epithelium; neuroprotective, improves cognition, increases brain neuronal stem cells, and enhances brain neuronal plasticity [13,67]. Expression inhibited in AD [51]. Expressed in human and rodent hypothalamus [16].	Receptor agonists reduce alcohol consumption in obese people [61].
**Insulin**	*INS*	Reduces blood glucose; regulates carbohydrate and lipid metabolism by increasing cell permeability to monosaccharides, amino acids, and fatty acids; accelerates the pentose phosphate cycle and glycogen synthesis in the liver [68,69]	Insulin signaling pathways are disrupted by ethanol feeding; alcohol-related brain damage is associated in insulin resistance in neurons; receptor gene depletion impairs survival and growth of cerebellar neurons; insulin sensitizers prevent alcohol-related brain damage and neurobehavioral dysfunction [39]; alcohol-related white matter pathology is associated with reduced insulin/IGF pathway signaling [70].
**Leptin**	*LEP*	Important regulator of energy balance by inhibiting food intake and promoting energy expenditure; helps regulate fat depots. Binds to leptin receptors in the	Inhibited in the frontal lobe by chronic alcohol feeding in an experimental model [19,50]. Leptin also reduced in

		brain to inhibit feeding and promote energy expenditure. Circadian rhythm disruption inhibits expression in the brain [71]; AD inhibits expression in brain (frontal lobe) with greater effects associated with AD severity and ApoE-ε4 genotype [51]	patients with fetal alcohol spectrum disorder [45].
**PP**	*NYP*/Pancreatic Polypeptide	Neuropeptide Y family member peptide; In the brain, PP is NPY, which is abundant in the brainstem, thalamus, hypothalamus, subcortical nuclei, and cerebral cortex. Stress driver via central nervous system actions counteracting excitatory effects of neuropeptide corticotropin-releasing factor [72]. Expressed in brain regions that regulate food intake, feal, learning, memory and anxiety [73]. Potent stimulator of food intake [48]; stimulates contraction of the basilar artery, which supplies blood to the posterior circulation in the brain including cerebellum, brainstem and thalamus.	Binge alcohol-mediated increases NPY in the prefrontal cortex and nucleus accumbens during adolescence, decreases weight gain, motivation, and active psychosocial stress coping [74]. Alcohol dependence increases NPY system plasticity and recruitment in the central amygdala [75].
**PYY**	*Peptide YY*	Member of the neuropeptide Y (NPY) family of peptides; signals the brain to attenuate food intake, anxiety, and depression-related behavior; Gut-brain axis; postprandial secretion targets the cortex and hypothalamus and inhibits dopamine release in the hypothalamus.	Alcohol did not inhibit serum levels (secretion) of PYY in normal adult subjects. Fetal alcohol spectrum disorder was not associated with reduced PYY in serum [45]

References

63. Calissendorff, J.; Danielsson, O.; Brismar, K.; Rojdmark, S. Alcohol ingestion does not affect serum levels of peptide YY but decreases both total and octanoylated ghrelin levels in healthy subjects. *Metabolism* **2006**, *55*, 1625–1629, doi:10.1016/j.metabol.2006.08.003.
64. Leggio, L.; Zywiak, W.H.; Fricchione, S.R.; Edwards, S.M.; de la Monte, S.M.; Swift, R.M.; Kenna, G.A. Intravenous ghrelin administration increases alcohol craving in alcohol-dependent heavy drinkers: a preliminary investigation. Biol Psychiatry 2014, 76, 734–741, doi:10.1016/j.biopsych.2014.03.019.
65. Hong, S.H.; Choi, K.M. Gut hormones and appetite regulation. Curr Opin Endocrinol Diabetes Obes 2024, 31, 115–121, doi:10.1097/MED.0000000000000859.
66. Baggio, L.L.; Drucker, D.J. Biology of incretins: GLP-1 and GIP. *Gastroenterology* **2007**, *132*, 2131–2157, doi:10.1053/j.gastro.2007.03.054.
67. Holscher, C. The incretin hormones glucagonlike peptide 1 and glucose-dependent insulinotropic polypeptide are neuroprotective in mouse models of Alzheimer's disease. Alzheimers Dement 2014, 10, S47–54, doi:10.1016/j.jalz.2013.12.009.
68. Khalid, M.; Alkaabi, J.; Khan, M.A.B.; Adem, A. Insulin Signal Transduction Perturbations in Insulin Resistance. Int J Mol Sci 2021, 22, 8590, doi:10.3390/ijms22168590.
69. Le, T.K.C.; Dao, X.D.; Nguyen, D.V.; Luu, D.H.; Bui, T.M.H.; Le, T.H.; Nguyen, H.T.; Le, T.N.; Hosaka, T.; Nguyen, T.T.T. Insulin signaling and its application. Front Endocrinol (Lausanne) 2023, 14, 1226655, doi:10.3389/fendo.2023.1226655.

70. de la Monte, S.M.; Tong, M. Molecular and biochemical pathologies in human alcohol-related cerebellar white matter degeneration. Adv Drug Alcohol Res 2025, 5, 15342, doi:10.3389/adar.2025.15342.
71. Karatsoreos, I.N.; Bhagat, S.; Bloss, E.B.; Morrison, J.H.; McEwen, B.S. Disruption of circadian clocks has ramifications for metabolism, brain, and behavior. Proceedings of the National Academy of Sciences of the United States of America 2011, 108, 1657–1662, doi:10.1073/pnas.1018375108.
72. Yerraguntla, H.; Onyekachi, J.; Giacometti, L.L.; Goldberg, S.L.; Barson, J.R.; Barker, J.M.; Reyes, B.A.S. Subcellular interactions of neuropeptide Y and corticotropin-releasing factor in the central nucleus of the amygdala in the mouse. bioRxiv 2025, doi:10.1101/2025.09.19.677477.
73. Tanaka, M.; Yamada, S.; Watanabe, Y. The Role of Neuropeptide Y in the Nucleus Accumbens. Int J Mol Sci 2021, 22, doi:10.3390/ijms22147287.
74. Brancato, A.; Castelli, V.; Cannizzaro, C.; Tringali, G. Adolescent binge-like alcohol exposure dysregulates NPY and CGRP in rats: Behavioural and immunochemical evidence. Prog Neuropsychopharmacol Biol Psychiatry 2023, 123, 110699, doi:10.1016/j.pnpbp.2022.110699.
75. Gilpin, N.W. Neuropeptide Y (NPY) in the extended amygdala is recruited during the transition to alcohol dependence. Neuropeptides 2012, 46, 253–259, doi:10.1016/j.npep.2012.08.001.
